# THE IL-6/IL-10 INFLAMMATORY AXIS AND PROGNOSIS OF CIRRHOSIS-ASSOCIATED HEPATOCELLULAR CARCINOMA: A NARRATIVE REVIEW

**DOI:** 10.1590/S0004-2803.24612025-163

**Published:** 2026-06-05

**Authors:** Rubens Barbosa REZENDE

**Affiliations:** 1 Universidade Federal de São Paulo (UNIFESP), Departamento de Biociências, Santos, SP, Brasil.

**Keywords:** Hepatocellular carcinoma, liver cirrhosis, interleukin-6, interleukin-10, biomarkers, prognosis, cytokines, survival, Biomarcadores, carcinoma hepatocelular, citocinas, cirrose hepática, interleucina-6, interleucina-10, prognóstico, sobrevida

## Abstract

**Background:**

Hepatocellular carcinoma (HCC) arises predominantly in cirrhotic livers and remains one of the leading causes of mortality related to chronic liver disease. Chronic inflammation, immune dysfunction, and tissue remodeling sustain hepatocarcinogenesis, making circulating cytokines promising candidates for clinical biomarkers.

**Objective:**

To critically synthesize evidence on the role of interleukin-6 (IL-6) and interleukin-10 (IL-10) as biomarkers associated with HCC occurrence, staging, therapeutic response, and prognosis in individuals with cirrhosis.

**Methods:**

A narrative review was performed based on a structured literature search in PubMed/MEDLINE, SciELO, Europe PMC, and publishers’ databases (2010-2025), using descriptors related to hepatocellular carcinoma, cirrhosis, IL-6, IL-10, prognosis, and biomarkers. Clinical studies assessing serum or plasma cytokine levels, meta-analyses, mechanistic reviews, and contemporary clinical guidelines were prioritized.

**Results:**

The majority of clinical studies indicate a consistent association between elevated IL-6 levels and poor prognosis, increased tumor burden, systemic inflammation, and inferior outcomes following both systemic and locoregional therapies. For IL-10, the evidence supports elevated levels in a substantial proportion of patients with HCC, with signals of association with tumor-related immunosuppression and worse outcomes in advanced disease, although some studies suggest a context- and disease-stage-dependent role.

**Conclusion:**

IL-6 demonstrates greater consistency as a biomarker of progression and prognosis in cirrhosis-associated HCC, whereas IL-10 emerges as an immune regulatory marker with heterogeneous behavior depending on disease etiology, tumor stage, and the tumor microenvironment. Standardization of assay methodologies, cutoff values, and multivariable prognostic models is essential for clinical implementation.

## HIGHLIGHTS

• This narrative review critically synthesizes evidence on the role of IL-6 and IL-10 as inflammatory biomarkers in cirrhosis-associated hepatocellular carcinoma.

• A structured literature search was conducted, with qualitative and comparative analysis of key clinical, mechanistic, and prognostic studies.

• The findings indicate a consistent association between elevated IL-6 levels, tumor progression, impaired liver function, and reduced survival, whereas IL-10 exhibits context- and stage-dependent behavior.

• IL-6 emerges as a robust prognostic biomarker, while IL-10 may add value within multimarker panels and integrated prognostic models.

## RESUMO

**Contexto:**

O carcinoma hepatocelular (CHC) emerge majoritariamente em fígados cirróticos e permanece como uma das principais causas de mortalidade relacionada a doença hepática crônica. A inflamação crônica, a disfunção imune e a remodelação tecidual sustentam a hepatocarcinogênese, tornando citocinas circulantes candidatas a biomarcadores clínicos.

**Objetivo:**

Sintetizar criticamente evidências sobre o papel da interleucina-6 (IL-6) e da interleucina-10 (IL-10) como biomarcadores associados a ocorrência, estadiamento, resposta terapêutica e prognóstico do CHC em indivíduos com cirrose.

**Métodos:**

Revisão narrativa baseada em busca estruturada em PubMed/MEDLINE, SciELO, Europe PMC e bases de editoras (2010-2025), utilizando descritores relacionados a hepatocellular carcinoma, cirrhosis, IL-6, IL-10, prognosis e biomarkers. Foram priorizados estudos clínicos com dosagem sérica/plasmática, metanálises, revisões mecanísticas e diretrizes contemporâneas.

**Resultados:**

A maioria dos estudos indica associação entre IL-6 elevada e pior prognóstico, maior carga tumoral, inflamação sistêmica e desfechos inferiores em terapias sistêmicas e locorregionais. Para IL-10, as evidências sustentam elevação em uma fração relevante de pacientes com CHC, com sinal de associação a imunossupressão tumoral e piores desfechos em doença avançada, embora alguns trabalhos sugiram papel dependente de contexto e fase da doença.

**Conclusão:**

IL-6 apresenta consistência maior como biomarcador de progressão e prognóstico no CHC sobre cirrose, enquanto IL-10 se destaca como marcador de regulação imune com comportamento heterogêneo conforme etiologia, estadiamento e microambiente tumoral. A padronização de ensaios, pontos de corte e modelos multivariados é essencial para adoção clínica.

## INTRODUCTION

Hepatocellular carcinoma (HCC) is the most common primary malignancy of the liver and ranks among the leading causes of cancer-related mortality worldwide. Its high lethality is largely attributable to late diagnosis and to the complex interaction between tumor-related, inflammatory, and hepatic functional factors. It is estimated that more than 80% of HCC cases arise in previously cirrhotic livers, highlighting liver cirrhosis as the main pathological substrate for tumor development^1^ ^,^ ^2^.

Cirrhosis is characterized by a state of persistent chronic inflammation, progressive tissue remodeling, and immune dysfunction, creating a microenvironment conducive to hepatocarcinogenesis. Within this framework, continuous activation of inflammatory pathways and sustained release of soluble mediators play a central role in malignant transformation of hepatocytes, tumor angiogenesis, and immune surveillance evasion. Thus, systemic inflammation emerges not only as an etiopathogenic factor but also as a prognostic determinant in HCC^3^ ^,^ ^4^.

Among the inflammatory mediators involved in this process, the cytokines IL-6 and IL-10 have been extensively investigated due to their immunomodulatory functions and direct effects on the hepatic tumor microenvironment. IL-6 is recognized as a pleiotropic pro-inflammatory cytokine capable of activating the JAK/STAT3 signaling pathway, thereby promoting cell proliferation, resistance to apoptosis, and angiogenesis-mechanisms strongly implicated in HCC progression^5^ ^,^ ^6^. Accumulating clinical evidence suggests a consistent association between elevated serum IL-6 levels, increased tumor burden, and reduced overall survival in patients with cirrhosis-associated HCC^7^ ^,^ ^8^.

Conversely, IL-10 plays a predominantly immunoregulatory role, acting to suppress excessive inflammatory responses and modulate the activity of antigen-presenting cells and T lymphocytes. In the tumor context, however, this immunosuppressive property may facilitate immune escape and neoplastic progression. Clinical studies have reported elevated IL-10 levels in a substantial proportion of patients with HCC, particularly in more advanced stages, although its effects appear to depend on disease stage, cirrhosis etiology, and interactions with other inflammatory pathways^9^ ^,^ ^10^.

Despite the growing body of studies on IL-6 and IL-10, the literature remains heterogeneous with respect to methodological designs, evaluated populations, laboratory cutoff values, and analyzed clinical outcomes. Moreover, most available evidence derives from observational studies, which hampers integrated interpretation and translation of findings into clinical practice. Under these circumstances, a critical synthesis becomes relevant to organize existing knowledge, identify consistent patterns, and highlight research gaps, particularly in light of recent advances in systemic and immunomodulatory therapies for HCC^11^ ^,^ ^12^.

Accordingly, the present study aimed to critically synthesize available evidence on the role of IL-6 and IL-10 as biomarkers associated with HCC occurrence, staging, therapeutic response, and prognosis in patients with liver cirrhosis. The rationale for this work lies in the need to integrate clinical, mechanistic, and prognostic data, contributing to a clearer understanding of the IL-6/IL-10 inflammatory axis in HCC and providing support for the development of more accurate prognostic models and for future translational research focused on incorporating inflammatory biomarkers into clinical practice.

## METHODS

### Study design

The present manuscript consists of a narrative literature review, an approach recommended when the primary objective is to critically integrate clinical, mechanistic, and prognostic evidence, allowing conceptual contextualization, translational discussion, and identification of research gaps^13^ ^-^ ^16^.

### Search strategy and information sources

The literature search was performed in a structured and reproducible manner across PubMed/MEDLINE, SciELO, and Europe PMC, complemented by manual searches of reference lists from key articles and international clinical guidelines. The search period included publications from 2010 to 2025, prioritizing recent and clinically relevant literature. Controlled descriptors and free-text terms were combined using Boolean operators, including: “hepatocellular carcinoma,” “cirrhosis,” “interleukin-6” OR “IL-6,” “interleukin-10” OR “IL-10,” “biomarkers,” “prognosis,” “survival,” and “BCLC.”

### Eligibility criteria

Eligible studies included observational clinical studies, prospective or retrospective cohorts, clinical trials, systematic reviews, meta-analyses, and clinical guidelines that assessed serum or plasma levels of IL-6 and/or IL-10 in patients with hepatocellular carcinoma, preferably in the context of liver cirrhosis, and examined associations with tumor staging, therapeutic response, or survival outcomes. Case reports, exclusively experimental studies without direct clinical correlation, articles lacking adequate methodological description, or studies that did not report relevant clinical outcomes were excluded.

### Evidence synthesis and analysis

Evidence synthesis was performed in a qualitative and interpretative manner, with extraction of the main methodological and clinical data from the selected studies. The included articles were compared in terms of study design, population characteristics, cytokines assessed, principal findings, and clinical outcomes, and the results were organized in the form of a comparative table.

### Classification of level of evidence and critical appraisal of studies

In addition, the included studies were classified according to level of evidence in order to critically qualify the methodological robustness of the available data. The categorization of level of evidence was performed qualitatively, based on study design and on hierarchies of evidence widely employed in health research, inspired by the principles of the GRADE system and by methodological recommendations for narrative reviews.

Systematic reviews and meta-analyses were classified as level I; analytical observational studies, such as well-defined prospective or retrospective cohorts, were classified as level II; and cross-sectional or exploratory observational studies were classified as level III.

This classification was not intended to be exclusionary but rather served as an analytical framework to contextualize the strength of the evidence, critically interpret the results, and explicitly identify limitations inherent to each study design. Levels of evidence, together with critical observations regarding potential biases and methodological limitations, were systematically presented in a comparative table, as recommended for narrative reviews with a clinical-translational focus^13^ ^-^ ^16^.

The limitations inherent to the narrative review design include the absence of quantitative synthesis and the potential risk of selection bias, which were mitigated in the present study through a structured search strategy, diversity of information sources, and critical appraisal of the level of evidence.

## RESULTS

The chronological synthesis of the studies included in this narrative review is presented in Table 1, which summarizes methodological design, population characteristics, cytokines evaluated, main findings, clinical outcomes, level of evidence, and critical limitations. This comparative organization allows an integrated visualization of the evolution of evidence regarding the prognostic role of IL-6 and IL-10 in cirrhosis-associated hepatocellular carcinoma, as well as the identification of consistent and divergent findings over time.

**TABLE 1:** Methodological characteristics, main findings, and level of evidence of studies on IL-6 and IL-10 in cirrhosis-associated hepatocellular carcinoma.

Author/Year	Study design and population	Comparator	Cytokine(s)	Main findings	Outcomes	Level of evidence	Critical appraisal (bias/limitations)
Chau et al., 2000^7^	Cohort; resectable HCC	High vs low IL-10	IL-10 (and IL-6)	IL-10 (but not IL-6) was associated with clinical outcomes in resectable HCC	Recurrence/overall survival (OS)	III	Single-sample study; resectable population; lack of robust multivariable adjustment
Hattori et al., 2003^9^	Cohort; unresectable HCC	Stratification by IL-10 levels	IL-10	IL-10 was associated with poorer prognosis and reduced antitumor immunity	Survival and immune parameters	III	Older observational study; small sample size; non-standardized laboratory techniques
Chan et al., 2012^8^	Prospective cohort; unresectable HCC (n=222)	IL-10 >1 pg/mL vs ≤1 pg/mL	IL-10	High IL-10 levels were associated with worse overall survival and retained independent prognostic value	OS (adjusted hazard ratio)	II	Well-defined cohort, but single-center; potential selection bias
Loosen et al., 2018^17^	Cohort; HCC under systemic therapy	Prognostic cutoff	IL-6 (and IL-8)	High IL-6 levels were associated with reduced overall survival; IL-6/IL-8 acted as prognostic stratifiers	OS	II	Retrospective cohort; therapeutic heterogeneity
Shakiba et al., 2018^24^	Systematic review/meta-analysis	HCC vs controls/hepatitis/cirrhosis	IL-10	Serum IL-10 levels were increased in HCC, suggesting complementary diagnostic value	Standardized mean difference	I	Meta-analysis; methodological heterogeneity among included studies
Öcal et al., 2021^18^	Post hoc analysis; SORAMIC trial (n = 83), Y-90 radioembolization plus sorafenib	IL-6 cutoff: 6.53 pg/mL	IL-6 (and IL-8)	High baseline IL-6 was the only independent factor for overall survival and was also associated with hepatic dysfunction	OS and hepatic dysfunction	II	Post hoc analysis; population selected for radioembolization
Chamseddine et al., 2023^10^	Clinical cohort; HCC (multiple stages)	Stratification by circulating levels	IL-6, IL-10 and other interleukins	Elevated IL-6 and IL-10 levels were associated with inferior overall survival	OS (Kaplan-Meier analysis; multivariable models)	II	Observational cohort; assessment of multiple cytokines increases risk of confounding
Yang et al., 2023^19^	Cohort; unresectable HCC receiving systemic therapy (Atezolizumab + Bevacizumab)	High vs low IL-6	IL-6	High IL-6 levels were associated with poorer outcomes and immune dysfunction	Overall survival/progression-free survival (OS/PFS) and treatment response	II	Population receiving a specific systemic therapy; limited generalizability
Lin et al., 2024^20^	Phase II trial/clinical study; advanced HCC with cytokine monitoring	Cutoff values and dynamics	IL-6	Higher IL-6 levels were associated with shorter overall survival; reductions in IL-6 were accompanied by improvements in clinical indicators	OS/PFS (hazard ratio by cutoff)	II	Short follow-up; lack of external validation
Hu et al., 2024^21^	HCC receiving radiotherapy	IL-6 >7.8 pg/mL vs ≤7.8 pg/mL	IL-6	High pre-radiotherapy IL-6 levels were associated with worse prognosis	OS/PFS (adjusted hazard ratio)	II	Cutoff defined a posteriori; potential inflammatory bias
Faria et al., 2025^23^	Cross-sectional study; cirrhosis (n=21) vs cirrhosis + HCC (n=26); BCLC A/B	Cirrhosis without HCC	IL-6, IL-10	IL-6 levels were higher in BCLC stage B, whereas IL-10 levels were higher in BCLC stage A; AST and ALT levels were higher in HCC	Reduced survival in HCC; IL-6/IL-10 suggest disease progression	III	Cross-sectional study; does not allow causal inference; limited sample size
Miura et al., 2025^22^	Derivation and validation study; HCC treated with Atezolizumab + Bevacizumab vs Lenvatinib	Groups according to baseline IL-6 levels	IL-6	Elevated IL-6 was an independent predictor of worse prognosis, while low IL-6 levels were associated with improved progression-free and overall survival	OS/PFS	II	Requires multicenter validation; influence of prior therapy

Aspartate aminotransferase (AST); alanine aminotransferase (ALT).

Source: Prepared by the author, 2025.

Across the included studies, elevated circulating IL-6 levels were consistently associated with worse overall survival (OS) and progression-free survival (PFS), increased tumor burden, impaired liver function, and unfavorable outcomes across different therapeutic modalities, including locoregional therapies, radiotherapy, systemic therapy, and immunotherapy^7^ ^,^ ^8^ ^,^ ^17^ ^-^ ^23^.

In contrast, IL-10 demonstrated a more heterogeneous pattern across studies. Elevated IL-10 levels were reported in a significant proportion of patients with hepatocellular carcinoma, particularly in advanced disease, while other studies suggested higher IL-10 levels in earlier stages, possibly reflecting immunoregulatory mechanisms. The prognostic significance of IL-10 varied according to tumor stage, cirrhosis etiology, and clinical context^7^ ^-^ ^10^ ^,^ ^23^.

**FIGURE 1:**
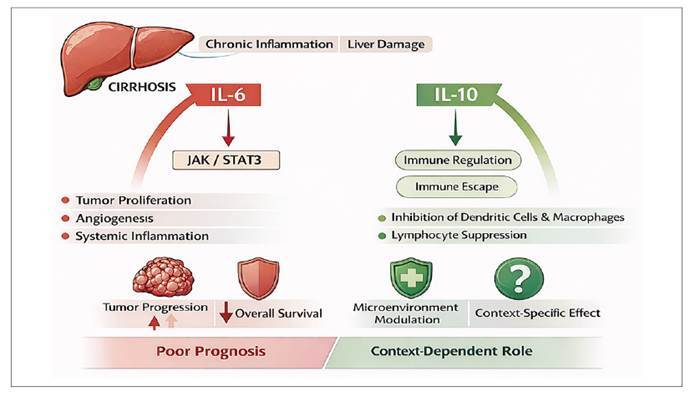
Integrated overview of the IL-6/IL-10 inflammatory axis and its prognostic implications in cirrhosis-associated hepatocellular carcinoma.

Source: prepared by the author using Python (Matplotlib library), 2025.

**FIGURE 2:**
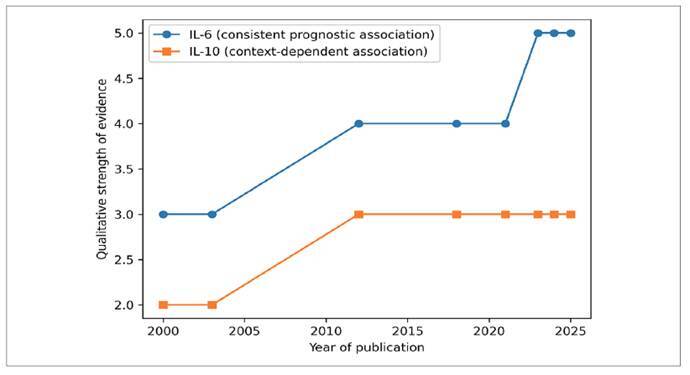
Qualitative synthesis of the evidence on the prognostic role of interleukins IL-6 and IL-10 in cirrhosis-associated hepatocellular carcinoma.

Source: prepared by the author using Python software (Matplotlib library), 2025.

The biological and prognostic framework of the IL-6/IL-10 inflammatory axis in cirrhosis-associated hepatocellular carcinoma is summarized in Figure 1, whereas the temporal evolution of the strength of evidence reported in the literature is qualitatively synthesized in Figure 2.

Studies conducted in contemporary therapeutic settings, including patients treated with immune checkpoint inhibitors and combination regimens, demonstrated that baseline IL-6 levels retained prognostic value even in the era of immunotherapy^19^ ^-^ ^22^. Some studies further suggested that combined evaluation of IL-6 and IL-10 may enhance prognostic stratification compared with isolated cytokine assessment^18^ ^,^ ^10^ ^,^ ^23^.

## DISCUSSION

With methodological advances, cohort studies conducted in the subsequent decade, such as that by Chan et al. (2012^8^, began to integrate clinical and laboratory variables, demonstrating that IL-6 not only reflects systemic inflammation but is also independently associated with tumor progression and reduced overall survival. These findings reinforce the hypothesis that IL-6 acts as a central mediator of JAK/STAT3 pathway activation within the hepatic tumor microenvironment^8^.

In addition to its prognostic value, the consistency of the IL-6 prognostic signal across different therapeutic settings is compatible with its direct involvement in oncogenic pathways (IL-6/JAK/STAT3), induction of C-reactive protein, promotion of angiogenesis, and remodeling of the tumor microenvironment. IL-6 is also associated with sarcopenia, cachexia, and systemic inflammation, components that worsen prognosis independently of tumor burden. Thus, IL-6 tends to capture both biological aggressiveness and host clinical vulnerability^24^ ^-^ ^26^.

Beyond its prognostic role, IL-6 may contribute to risk stratification in locoregional treatment strategies (such as radiotherapy and radioembolization) and to response assessment when combined with classical markers (alpha-fetoprotein [AFP]) and functional scores (ALBI/Child-Pugh). Nevertheless, challenges remain, including variability in assay methodologies, heterogeneous cutoff values, and the influence of infections, hepatic decompensation, and extrahepatic inflammation^18^ ^,^ ^21^.

From a translational perspective, IL-6 may be explored in prognostic panels alongside AFP, ALBI/Child-Pugh scores, and inflammatory markers (C-reactive protein [CRP], neutrophil-to-lymphocyte ratio [NLR]). However, prior to routine clinical adoption, several requirements must be addressed: (i) external validation in multicenter cohorts with assay standardization; (ii) definition of reproducible cutoff values; and (iii) assessment of temporal stability and the impact of intercurrent events (infections, bleeding, hepatic decompensation)^7^ ^-^ ^10^ ^,^ ^17^ ^,^ ^24^ ^,^ ^26^ ^,^ ^28^.

Studies such as those by Loosen et al. (2018)^17^ and Shakiba et al. (2018)^24^ expanded this understanding by demonstrating, through multicenter cohorts and meta-analyses, that elevated IL-6 levels remain consistently associated with unfavorable outcomes, regardless of the therapeutic modality employed. Nevertheless, the methodological heterogeneity observed among studies included in these meta-analyses highlights the need for caution when extrapolating these results.

Overall, elevated IL-6 levels are associated with poorer prognosis and greater tumor aggressiveness in HCC. Observational studies and clinical cohorts report associations with reduced OS and/or PFS, even after adjustment for clinical covariates. In more contemporary therapeutic settings, high baseline IL-6 has been linked to worse outcomes in systemic treatments, including combination immunotherapy (e.g., atezolizumab plus bevacizumab), suggesting that IL-6 may reflect an inflammatory and immunological state unfavorable to therapeutic response^12^ ^,^ ^27^ ^,^ ^29^ ^,^ ^30^.

Recent investigations conducted in modern therapeutic contexts, such as those by Yang et al. (2023)^19^, Lin et al. (2024)^20^, and Hu et al. (2024)^21^, evaluated patients undergoing systemic therapy and immunotherapy and demonstrated that IL-6 retains prognostic value even in the era of immune checkpoint inhibitors. These findings suggest that systemic inflammation remains a relevant prognostic determinant, independent of therapeutic advances.

In parallel, IL-10 illustrates the paradox of regulatory cytokines in cancer: while reducing chronic inflammation and potentially limiting tumor-promoting stimuli, its inhibitory effects on macrophages and dendritic cells, as well as its reduction of effector T-lymphocyte activation, may favor immune escape. This dual role explains the heterogeneous results observed across studies and reinforces the need for contextual interpretation^10^ ^,^ ^17^ ^,^ ^26^ ^,^ ^28^.

Accordingly, IL-10 emerges as a cytokine with more complex behavior. Studies such as those by Öcal et al. (2021)^18^ and Chamseddine et al. (2023)^10^ suggest that elevated IL-10 levels may be associated with early-stage HCC, possibly reflecting an early immunoregulatory mechanism. Thus, composite metrics (e.g., TNF-α/IL-10; IL-6/IL-10 ratios) and integration with clinical data may be more informative than isolated IL-10 measurement. However, this same immunosuppressive property may promote tumor escape in more advanced contexts, which helps explain the variability of results observed across different populations.

Meta-analyses indicate that serum IL-10 levels tend to be higher in HCC compared with controls and some hepatitis/cirrhosis populations, reinforcing its potential role as a complementary biomarker. In advanced disease, both classical and contemporary studies point to elevated IL-10 as a factor associated with poorer prognosis, reduced hepatic reserve, and more advanced tumor staging^8^.

Nevertheless, available evidence also suggests dependence on disease phase and etiological context. In certain settings, IL-10 may increase early as a regulatory response and, in more advanced stages, decline due to immune exhaustion or predominance of pro-inflammatory pathways. Therefore, IL-10 may be better interpreted within biomarker panels (e.g., TNF-α/IL-10; IL-6/IL-10 ratios) and multivariable models rather than in isolation^7^ ^-^ ^10^ ^,^ ^17^ ^-^ ^23^.

The study by Miura et al. (2025)^22^ further contributes by proposing integrated prognostic models combining IL-6, IL-10, and clinical parameters, indicating that multimarker approaches may overcome the limitations of isolated biomarkers. Nonetheless, the need for multicenter external validation remains a relevant limitation.

Within this context, the cross-sectional study by Faria et al. (2025)^23^, included in the present review, compared isolated cirrhosis (n=21) with cirrhosis plus HCC (n=26) and demonstrated that IL-6 levels were significantly elevated in BCLC stage B HCC, whereas IL-10 levels were higher in BCLC stage A disease. The authors also observed higher AST and ALT levels in the HCC group and a marked reduction in survival among patients with neoplasia (median 331 days) compared with cirrhosis without tumor (median 1,838 days). This study reinforces the notion that cytokines may add prognostic information when interpreted alongside markers of hepatic function and nutritional status.

Moreover, it clearly synthesizes the relationship between inflammatory cytokines, nutritional status, and survival, reinforcing IL-6 as a marker of tumor progression and IL-10 as a potential indicator of early immune modulation. Despite the inherent limitations of the cross-sectional design, these findings converge with the international literature and strengthen the external consistency of the evidence analyzed^23^.

## CONCLUSION

This narrative review critically integrated the available evidence on the role of the inflammatory cytokines IL-6 and IL-10 in the progression of cirrhosis-associated hepatocellular carcinoma, highlighting systemic inflammation as a central axis in hepatic carcinogenesis and clinical prognosis. The synthesis of clinical, mechanistic, and prognostic data reinforces the relevance of inflammatory pathways in shaping tumor behavior and patient outcomes.

The chronological and comparative analysis of the included studies demonstrated a growing and consistent body of evidence supporting the prognostic value of IL-6. Elevated serum IL-6 levels were robustly and recurrently associated with tumor progression, impaired hepatic function, and reduced overall survival, regardless of clinical stage or therapeutic modality. This consistency across heterogeneous clinical settings underscores IL-6 as a marker that captures both tumor biological aggressiveness and host vulnerability.

In contrast, IL-10 exhibited a more heterogeneous and context-dependent behavior, suggesting a dual role in the natural history of hepatocellular carcinoma. Available evidence indicates that elevated IL-10 levels may reflect early immunoregulatory mechanisms in initial disease stages, whereas in more advanced settings its immunosuppressive activity may contribute to tumor immune escape. This variability highlights the importance of cautious and contextual interpretation, particularly in light of the predominantly observational nature of the available literature.

The critical appraisal of the level of evidence revealed recurrent methodological limitations across studies, including observational designs, limited sample sizes, etiological heterogeneity, and lack of standardization in cytokine assays and serum cutoff values. These factors limit direct comparability and reinforce the need for prudence in translating findings into clinical practice. Nevertheless, the convergence of results over time, especially regarding IL-6, confers external consistency and strengthens its candidacy as a prognostic biomarker.

Recent investigations further suggest that integrated prognostic models combining inflammatory biomarkers with established clinical and functional parameters may outperform isolated cytokine measurements. In this context, IL-6 emerges as a clinically promising biomarker for prognostic stratification and disease monitoring, whereas IL-10 represents a target of interest for future studies, particularly within multimarker panels and immunological frameworks that account for tumor microenvironment dynamics.

In light of these findings, future research should prioritize well-designed prospective and multicenter studies, robust multivariable analyses, and harmonization of laboratory methods. Such approaches are essential to validate current evidence, clarify underlying mechanisms, and enable the incorporation of inflammatory biomarker panels into routine clinical management. Overall, this review contributes to consolidating current knowledge and guiding future translational research in oncologic hepatology, with potential implications for prognostic stratification and clinical monitoring in patients with cirrhosis-associated hepatocellular carcinoma.

## Data Availability

not applicable - the study did not use research data
